# The relationship between hemoglobin glycation index and the risk of cardiovascular disease in populations with diabetes or prediabetes: a population-based cohort study

**DOI:** 10.1186/s13098-025-01754-0

**Published:** 2025-05-26

**Authors:** Zheng Wang, Fachao Shi, Long Wang, Caoyang Fang

**Affiliations:** 1 Department of Cardiology, The Second People’s Hospital of Hefei, Hefei Hospital, Affiliated to Anhui Medical University, Hefei, Anhui 230000 China; 2 Department of Cardiology, Maanshan People’s Hospital, Maanshan, Anhui 243000 China; 3 Department of Emergency, First Affiliated Hospital of University of Science and Technology of China, Anhui Provincial Hospital, Hefei, Anhui 230000 China

**Keywords:** Hemoglobin glycation index, HGI, Diabetes, Prediabetes, Cardiovascular disease, CVD

## Abstract

**Objective:**

The relationship between Glycated Hemoglobin Index (HGI) and cardiovascular disease (CVD) risk in individuals with diabetes or prediabetes remains unclear. Therefore, this study aims to investigate the relationship between baseline HGI and CVD risk in U.S. adults with diabetes or prediabetes.

**Methods:**

This study analyzed data from 10,889 diabetic or prediabetic participants from the National Health and Nutrition Examination Survey (NHANES). Weighted multivariable regression analysis and subgroup analyses were employed to assess the relationship between HGI and CVD risk. Restricted cubic splines were used to explore nonlinear associations, along with threshold effect analysis and subgroup analyses.

**Results:**

A total of 10,889 participants (mean age 52.82 years, 54.57% male) were included in this study. We observed a U-shaped relationship between HGI and the risk of cardiovascular disease (CVD) (P nonlinear < 0.0001), heart attack (P nonlinear = 0.0006), and congestive heart failure (CHF) (P nonlinear = 0.0001). The inflection points for HGI concerning CVD, heart attack, and CHF were − 0.140, -0.447, and − 0.140, respectively. When baseline HGI exceeded these thresholds, each unit increase in HGI was significantly associated with higher risks of CVD (OR: 1.34, 95% CI: 1.23–1.48), heart attack(OR: 1.34, 95% CI: 1.20–1.51), and CHF (OR: 1.39, 95% CI: 1.22–1.58).Subgroup analysis revealed significant differences in CHF risk associated with HGI across racial groups (interaction *P* = 0.03).

**Conclusion:**

In individuals with diabetes and prediabetes, HGI displays a U-shaped relationship with CVD, heart attack, and CHF risks, with threshold values of -0.14, -0.45, and − 0.14, respectively. HGI may serve as a more effective indicator for identifying populations at early risk for cardiovascular disease.

**Supplementary Information:**

The online version contains supplementary material available at 10.1186/s13098-025-01754-0.

## Introduction

Cardiovascular disease (CVD) is a leading cause of morbidity and disability, posing a significant threat to global public health. According to the 2019 Global Burden of Disease Study, the number of individuals affected by cardiovascular disease increased by 252 million from 1990 to 2019, and the cumulative death toll rose by 1.54 times [1, 2]. Stroke and ischemic heart disease dominate the spectrum of cardiovascular diseases, with years lived with disability (DALYs) and years of life lost (YLL) continuing to increase [3, 4]. Surprisingly, in some countries and regions, age-standardized incidence and mortality rates for cardiovascular disease began to rise and fall 30 years ago [5]. Therefore, timely assessment of cardiovascular disease risk is crucial for mitigating the burden of cardiovascular diseases.

Diabetes and its prediabetic state are increasingly serious public health issues globally and are recognized as significant risk factors for cardiovascular disease. Individuals with diabetes face a markedly increased risk of cardiovascular disease due to metabolic disturbances, chronic inflammation, and endothelial dysfunction caused by prolonged hyperglycemia [6, 7]. Studies indicate that the incidence of cardiovascular events in diabetes patients is more than double that of non-diabetic populations, highlighting the importance of early identification and intervention. Prediabetic individuals also face an elevated risk of cardiovascular disease; therefore, timely blood glucose monitoring and lifestyle interventions are crucial for reducing this risk [8].

The Hemoglobin Glycation Index (HGI) is a significant indicator that reflects individual variations in hemoglobin glycation tendency, first proposed by Hempe et al. in 1996 [9]. HGI is defined as the difference between measured glycated hemoglobin (HbA1c) and predicted HbA1c based on mean blood glucose levels [10, 11]. Physiologically, HGI reflects differences in hemoglobin glycation levels among individuals with similar blood glucose concentrations—a variation potentially influenced by multiple factors, including genetic background [12], erythrocyte lifespan [13], oxidative stress levels [14], and protein modification enzyme activity [15]. Research demonstrates that HGI can differ significantly even between individuals with comparable glycemic control, suggesting that HGI may serve as a risk marker independent of traditional glycemic indicators.Clinically, HGI’s importance manifests in several key aspects. First, it can identify individuals whose HbA1c values are inconsistent with their average blood glucose levels, helping clinicians more accurately assess glycemic control status [16]. Second, HGI may reflect susceptibility to tissue glycation damage, thereby correlating with risks of diabetic complications [17]. Furthermore, HGI can potentially influence diabetes diagnosis and treatment decisions based on HbA1c measurements [18].

In recent years, numerous studies both in China and abroad have explored the association between the hemoglobin glycation index (HGI) and cardiovascular disease risk. In China, research has demonstrated that a high HGI is significantly associated with an increased risk of adverse cardiovascular events [19]. Similarly, another study found that HGI is closely related to long-term outcomes in patients with coronary artery disease following percutaneous coronary intervention (PCI), indicating that HGI may serve as a predictor of long-term mortality risk in patients undergoing PCI [20].

Hempe et al.‘s analysis of data from the ACCORD trial revealed that patients with high HGI benefited more from intensive glycemic control, whereas patients with low HGI paradoxically experienced increased mortality risk in the intensive treatment group [11]. This finding emphasizes HGI’s potential value in guiding personalized treatment decisions. Additionally, a long-term follow-up study conducted by Rodriguez-Segade et al. indicated that for diabetic patients, each 1% increase in HGI was associated with a 14% increase in cardiovascular event risk [21].

In Europe, studies by Nayak et al. from the United Kingdom confirmed a positive correlation between HGI and the risk of diabetic nephropathy and retinopathy [22].In South Korea, Kim et al. reported long-term associations between HGI and both microvascular and macrovascular complications of diabetes, suggesting that elevated HGI may serve as an independent predictor of adverse outcomes in diabetes [23].

Nevertheless, considerable controversy persists regarding the relationship between HGI and cardiovascular disease risk in patients with diabetes or prediabetes. Our objective is to determine whether HGI may have significant predictive value for cardiovascular disease likelihood among American patients with diabetes or prediabetes.

## Methods

### Study design and population

The National Health and Nutrition Examination Survey (NHANES) is an important research program aimed at assessing the health and nutritional status of adults and children residing in the United States. This program is implemented by the Centers for Disease Control and Prevention (CDC), which is responsible for providing national health statistics. The NHANES protocols have been approved by the CDC’s Institutional Review Board, and all participants have provided informed consent to protect their rights. Therefore, the Ethics Committee of the Second People ‘s Hospital of Hefei exempted this ethical application. Additionally, the dataset generated from this research can be accessed through the NHANES official website: https://www.cdc.gov/nchs/nhanes/index.html.

**Fig. 1:**
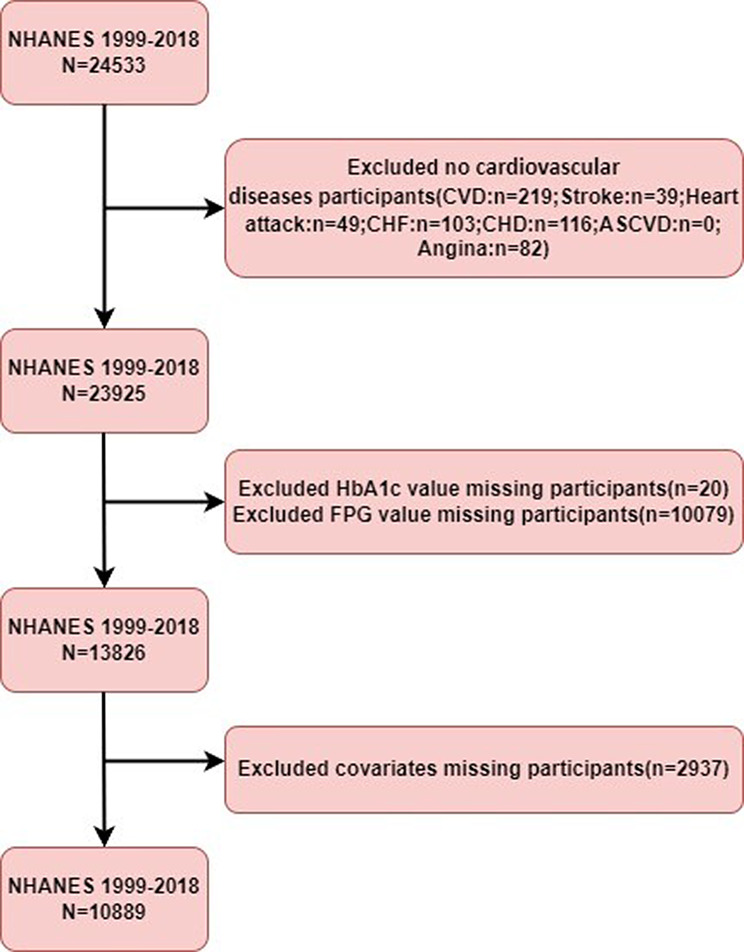
Study Flow Chart

This study is based on publicly available data from the National Health and Nutrition Examination Surveys (NHANES) from 1999 to 2018. Following the definitions from the American Diabetes Association (ADA), we selected adults aged ≥ 18 years old and determined diabetes or prediabetes status based on self-reported medical history or laboratory test results (fasting blood glucose, glycated hemoglobin, or oral glucose tolerance test). Inclusion criteria: ① Age ≥ 18 years; ② A clear diagnosis of diabetes or prediabetes, with specific criteria including: fasting blood glucose ≥ 7.0 mmol/L or 2-hour oral glucose tolerance test level ≥ 11.1 mmol/L; random blood glucose ≥ 11.1 mmol/L; glycated hemoglobin (HbA1c) ≥ 6.5%; use of diabetes medications or insulin or a doctor’s diagnosis of diabetes; fasting blood glucose between 5.6 and 7 mmol/L or 2-hour oral glucose tolerance test level between 7.8 and 11 mmol/L; glycated hemoglobin (HbA1c) ≥ 5.7% and < 6.5%; or a doctor’s diagnosis of prediabetes; ③ Complete key laboratory data such as HbA1c, fasting blood glucose, and major covariate information. Exclusion criteria: ① Age < 18 years; ② Missing data for HbA1c, fasting blood glucose, or major covariates; ③ No recorded outcomes for cardiovascular disease. The final analytical sample consisted of 10,889 participants (Fig. 1).

### Hemoglobin glycosylation index (HGI) calculation

**Fig. 2:**
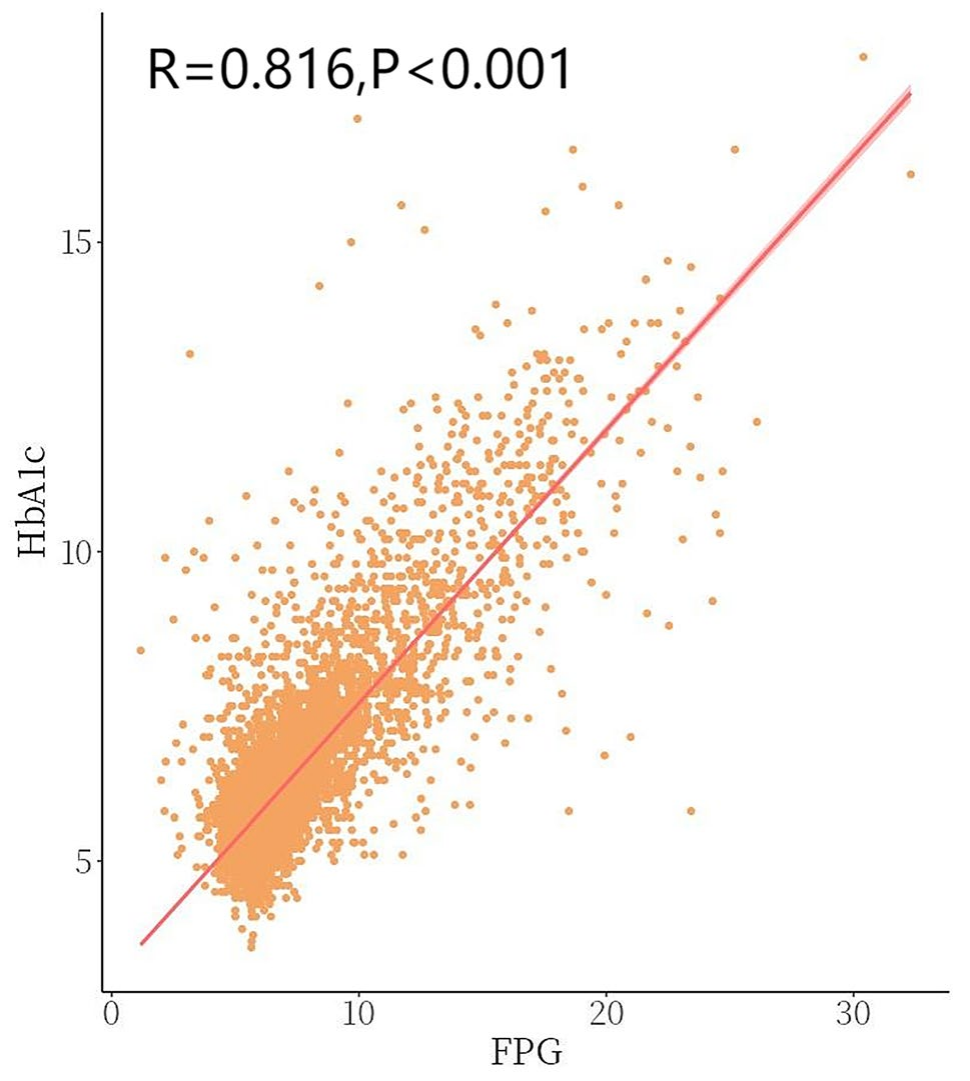
The correlation between HGI and HbA1c

This study utilized the NHANES database to analyze the relationship between HbA1c and fasting plasma glucose (FPG) levels in patients with diabetes and prediabetes. A linear relationship was established, resulting in the regression equation: predicted HbA1c = 0.442 FPG + 3.124 (Fig. 2). The study predicted the HbA1c levels of participants by substituting their FPG values into the regression equation. To assess the accuracy of this prediction, Hemoglobin Glycation Index (HGI) was used, calculated based on the difference between the actual HbA1c and the predicted HbA1c.

### Assessment of diabetes and prediabetes

This study only included patients with type 2 diabetes or prediabetes.The diagnostic criteria for diabetes include the following indicators: fasting blood glucose ≥ 7.0 mmol/L or a 2-hour oral glucose tolerance test result ≥ 11.1 mmol/L; random blood glucose ≥ 11.1 mmol/L; glycosylated hemoglobin (HbA1c) ≥ 6.5%; currently using diabetes medications or insulin, or confirmed by a physician to have diabetes. The criteria for prediabetes are: fasting blood glucose in the range of 5.6-7.0 mmol/L, or a 2-hour oral glucose tolerance test result between 7.8 and 11.0 mmol/L; glycosylated hemoglobin (HbA1c) ≥ 5.7% but < 6.5%; or diagnosed with prediabetes by a physician [24].This study utilized the structure of the NHANES database and the available clinical information, incorporating multiple parameters such as fasting blood glucose, level of glycated hemoglobin, physician-reported past diagnoses, and random blood glucose to assess diabetes and prediabetes. Since NHANES does not systematically collect acute symptoms related to diabetes, we can only rely on laboratory test results and past diagnoses as reference criteria for differentiation. This approach is consistent with common practices used in large-scale epidemiological studies based on real-world data in NHANES.

### Assessment of outcome variables

Health status assessment was based on standardized questionnaire data from the National Health and Nutrition Examination Survey (NHANES). The primary cardiovascular endpoints included cardiovascular disease (CVD), congestive heart failure (CHF), coronary heart disease (CHD), atherosclerotic cardiovascular disease (ASCVD), angina pectoris, heart attack (also known as myocardial infarction), and stroke.Disease identification methods were as follows: CHF was determined through questionnaire item MCQ160B, specifically asking whether “a healthcare professional has ever informed you of having congestive heart failure” [25]. CHD diagnosis followed previous NHANES research methodologies [26], confirmed by affirmative responses to the question “Has a healthcare professional ever informed you of having coronary heart disease?“.Angina history was assessed through self-reported data in the NHANES questionnaire file “MCQ.Doc,” with participants responding to whether they “had ever been informed of having angina pectoris” [27]. Heart attack (also known as myocardial infarction) was identified through household interview data variables, specifically affirmative responses to the question “Has a doctor ever informed you of having had a heart attack?” [28].Stroke was defined as previously diagnosed conditions self-reported during face-to-face interviews, specifically participants who answered “yes” to the question “Has a healthcare professional ever informed you that you have experienced a stroke?“ [29]. ASCVD history was based on affirmative responses to comprehensive inquiries about coronary heart disease/angina/myocardial infarction/stroke during the medical conditions section of household interviews [30].We defined CVD as a composite endpoint encompassing CHD, ASCVD, CHF, myocardial infarction, angina, and stroke. Additionally, secondary analyses were conducted for events associated with CHD, ASCVD, CHF, angina, stroke, and myocardial infarction [31, 32].

### Covariates

We included potential covariates that might affect the risk of cardiovascular disease in patients with diabetes or prediabetes. These variables included age, gender, race, education level, marital status, family income poverty ratio (PIR), hypertension, body mass index (BMI), and information on smoking and alcohol consumption.

Race categories included non-Hispanic Black, non-Hispanic White, Mexican American, and other races [33]. The family income poverty ratio (PIR) was divided into three levels: less than 1.3, 1.3 to 3.5, and above 3.5 [34]. Marital status was categorized as married, divorced, unmarried, and other [33]. Education level was classified as high school or below, high school or equivalent, and college or above [33]. Additionally, smoking status was defined as: (1) never smokers if they had smoked fewer than 100 cigarettes in their lifetime; (2) former smokers if they had smoked more than 100 cigarettes in their lifetime but currently do not smoke; (3) current smokers if they had smoked more than 100 cigarettes and smoke occasionally or daily [33]. Alcohol consumption was categorized into: never drinkers (fewer than 12 drinks in a lifetime), former drinkers (drank ≥ 12 times in the past year but not in the past year or drank < 12 times in a lifetime but ≥ 12 times), heavy drinking (≥ 3 drinks per day for females/≥4 drinks per day for males/binge drinking on 5 or more days per month), moderate drinking (≥ 2 drinks per day for females/≥3 drinks per day for males/binge drinking ≥ 2 days per month), and mild drinking (not including the above) [33]. Body mass index (BMI) was calculated as weight (kg) / height² (m²), with BMI < 25 kg/m² defined as normal, 25 kg/m² ≤ BMI ≤ 30 kg/m² defined as overweight, and BMI > 30 kg/m² defined as obese [31].Additionally, laboratory tests included creatinine, uric acid, blood urea nitrogen, neutrophils, lymphocytes, hemoglobin, and platelets.

### Statistical methods

This study utilized R software (version 4.3.2, available at https://www.r-project.org) for data analysis and followed the recommendations of the NHANES Analysis and Reporting Guidelines [35], taking into account the complex survey design and weights. In the weighted analysis, the MEC sample weights were used (WTMEC2YR/4 + WTMEC2YR/8). Participants were categorized into four groups based on quartiles of HGI (Q1-Q4). Continuous variables were presented as mean (SE), while categorical variables were reported as frequency % (SE). When comparing baseline characteristics between HGI quartiles, continuous variables were analyzed using one-way ANOVA, and categorical variables were assessed using the Pearson Chi-square test. A multivariable logistic regression model was employed to explore the relationship between HGI and the risk of cardiovascular disease in individuals with diabetes or prediabetes. Three models were established: Model 1 was unadjusted, Model 2 adjusted for age, race, and gender, and Model 3 further adjusted Model 2 for smoking, alcohol consumption, hypertension, and BMI. Additionally, a linear trend test was conducted by specifying the median HGI of each quartile as a continuous variable to evaluate trends.

To explore the dose-response relationship between HGI and the risk of cardiovascular disease in individuals with diabetes or prediabetes, we employed a logistic proportional hazards regression model using restricted cubic splines and smoothed curve fitting (penalized spline method). If a non-linear relationship was identified, we utilized piecewise linear regression to determine the inflection points. Additionally, we assessed the association between HGI and mortality through a segmented logistic proportional hazards model. Finally, subgroup analyses were conducted based on age, sex, race, BMI, PIR, and hypertension, aiming to reveal differences in the correlation between HGI and cardiovascular disease risk among different subgroups of individuals with diabetes or prediabetes. Results were considered statistically significant when p-values were less than 0.05 (two-tailed).

## Results

### Baseline characteristics of the study population

Table 1 presents the baseline characteristics of study participants stratified by HGI quartiles. A total of 10,889 participants were included in this study, with an average age of 52.82 years, of which 54.57% were male, and the prevalence of cardiovascular disease (CVD) was 12.52%. HGI was divided into four quartiles: Q1 (-7.678, -0.382), Q2 (-0.382, -0.054), Q3 (-0.054, 0.298), and Q4 (0.298, 9.483). Compared to the lowest quartile (Q1), patients in the higher quartiles of HGI had a significantly increased prevalence of CVD, coronary heart disease (CHD), congestive heart failure (CHF), atherosclerotic cardiovascular disease (ASCVD), heart attack, and stroke. Specifically, the prevalence of CVD in the quartiles was as follows: Q1: 9.98%, Q2: 11.19%, Q3: 12.60%, Q4: 18.08%; for CHD: Q1: 4.76%, Q2: 4.48%, Q3: 4.99%, Q4: 7.51%; for CHF: Q1: 2.25%, Q2: 3.03%, Q3: 3.39%, Q4: 6.55%; for ASCVD: Q1: 9.26%, Q2: 10.36%, Q3: 11.56%, Q4: 15.99%; for heart attack: Q1: 3.78%, Q2: 4.32%, Q3: 5.41%, Q4: 7.86%; for stroke: Q1: 3.06%, Q2: 3.88%, Q3: 3.56%, Q4: 5.54%; and for angina: Q1: 3.13%, Q2: 3.19%, Q3: 3.36%, Q4: 5.50%. Compared to participants in the lowest quartile, those in the higher quartiles of HGI were more likely to be non-Hispanic Black and obese, while having relatively lower educational attainment and household income.

**Table 1:** Baseline characteristics of the study population

Variables	Total	Q1(−7.678,−0.382)	Q2(−0.382,−0.054)	Q3(−0.054, 0.298)	Q4(0.298, 9.483)	*P*-value
**Age**,** yeaes**,** mean (SE)**	52.82(0.25)	48.57(0.44)	52.48(0.41)	55.59(0.39)	56.41(0.40)	< 0.0001
**Creatinine**,** umol/L**,** mean (SE)**	80.52(0.49)	81.24(0.71)	79.26(0.78)	78.37(0.66)	83.72(1.68)	0.002
**Uric acid**,** umol/L**,** mean (SE)**	342.80(1.23)	349.09(2.19)	342.48(1.92)	340.27(2.37)	336.81(2.43)	< 0.001
**BUN**,** mmol/L**,** mean (SE)**	5.12(0.03)	5.04(0.05)	5.04(0.05)	5.14(0.05)	5.34(0.06)	< 0.001
**Lymphocyte**,**×10**^**9**^**/L**,** mean(SE)**	2.03(0.01)	1.92(0.02)	2.00(0.02)	2.11(0.03)	2.18(0.03)	< 0.0001
**Neutrophils**,**×10**^**9**^**/L**,** mean(SE)**	4.18(0.03)	4.13(0.04)	4.15(0.04)	4.20(0.04)	4.25(0.05)	0.2
**Hemoglobin**,**×10**^**9**^**/L**,** mean(SE)**	14.50(0.03)	14.91(0.04)	14.64(0.04)	14.30(0.04)	13.93(0.05)	< 0.0001
**Platelet**,**×10**^**9**^**/L**,** mean(SE)**	248.53(0.92)	238.08(1.67)	249.69(1.55)	252.75(1.90)	257.65(1.89)	< 0.0001
**BMI**,**%(SE)**						< 0.0001
**< 25**	20.61(0.01)	21.37(1.03)	20.70(1.07)	20.95(1.04)	18.95(1.10)	
**25–30**	34.20(0.01)	38.41(1.14)	35.13(1.09)	31.15(1.02)	30.18(1.27)	
**> 30**	45.19(0.01)	40.22(1.33)	44.17(1.3)	47.90(1.12)	50.86(1.45)	
**Sex**,**%(SE)**						< 0.0001
Male	54.57(0.02)	65.38(1.16)	55.99(1.16)	47.32(1.24)	44.93(1.14)	
Female	45.43(0.01)	34.62(1.16)	44.01(1.16)	52.68(1.24)	55.07(1.14)	
**Race**,**%(SE)**						< 0.0001
Mexican American	8.42(0.01)	8.15(0.69)	8.59(0.84)	8.39(0.78)	8.64(0.79)	
Non-Hispanic Black	10.30(0.01)	5.25(0.44)	5.83(0.51)	12.28(0.86)	21.74(1.39)	
Non-Hispanic White	69.70(0.03)	76.69(1.19)	73.03(1.49)	68.55(1.56)	55.91(2.01)	
Other	11.58(0.01)	9.91(0.84)	12.55(0.87)	10.78(0.84)	13.71(1.08)	
**Marital**,**%(SE)**						< 0.0001
Married	60.20(0.02)	59.99(1/27)	61.88(1.43)	60.62(1.28)	57.68(1.37)	
Never Married	11.88(0.01)	15.11(1.08)	10.80(0.88)	9.18(0.68)	11.69(0.98)	
Divorced	10.80(0.01)	9.12(0.80)	11.33(0.84)	11.87(0.85)	11.30(0.80)	
Unmarried but have/had partner	17.13(0.01)	15.78(0.88)	15.99(0.90)	18.33(1.05)	19.34(0.99)	
**Education**,**%(SE)**						< 0.0001
Less than high School	18.92(0.01)	16.61(0.89)	18.07(0.98)	19.60(1.09)	22.78(1.13)	
High school or equivalent	25.58(0.01)	23.73(1.17)	24.97(1.18)	25.56(1.32)	29.23(1.30)	
College or above	55.50(0.02)	59.66(1.42)	56.96(1.47)	54.85(1.69)	47.99(1.26)	
**Smoke**,**%(SE)**						0.02
Never	49.94(0.01)	49.33(1.34)	50.09(1.24)	50.64(1.48)	49.81(1.56)	
Former	30.14(0.01)	32.15(1.32)	30.68(1.09)	29.60(1.20)	26.99(1.29)	
Now	19.92(0.01)	18.52(1.02)	19.23(1.01)	19.76(1.04)	23.20(1.27)	
**Alcohol**,**%(SE)**						< 0.0001
Never	11.54(0.01)	8.48(0.70)	10.26(0.84)	12.29(0.74)	17.02(1.04)	
Former	17.27(0.01)	12.87(0.86)	16.42(1.06)	18.72(0.87)	23.36(1.13)	
Mild	38.19(0.01)	38.37(1.38)	38.67(1.30)	40.43(1.44)	34.60(1.28)	
Moderate	14.71(0.01)	16.23(0.88)	16.34(0.88)	13.53(0.90)	11.54(0.84)	
Heavy	18.29(0.01)	24.04(1.00)	18.31(1.06)	15.02(0.98)	13.47(0.91)	
**Hypertension**,**%(SE)**						< 0.0001
Yes	49.90(0.01)	46.80(1.39)	44.13(1.23)	53.54(1.45)	58.23(1.31)	
No	50.10(0.01)	53.20(1.39)	55.87(1.23)	46.46(1.45)	41.77(1.31)	
**PIR**,**%(SE)**						< 0.0001
< 1.3	20.59(0.01)	19.06(1.00)	18.92(0.92)	21.16(1.09)	24.53(1.18)	
1.3–3.5	37.18(0.01)	33.92(1.39)	37.20(1.25)	37.67(1.31)	41.47(1.25)	
> 3.5	42.23(0.02)	47.02(1.58)	43.88(1.47)	41.17(1.66)	34.00(1.21)	
**CVD**,**%(SE)**						< 0.0001
Yes	12.52(0.01)	9.98(0.62)	11.19(0.75)	12.60(0.87)	18.08(0.97)	
No	87.48(0.02)	90.02(0.62)	88.81(0.75)	87.40(0.87)	81.92(0.97)	
**Stroke**,**%(SE)**						0.002
Yes	3.89(0.00)	3.06(0.40)	3.88(0.46)	3.56(0.40)	5.54(0.55)	
No	96.11(0.02)	96.40(0.40)	96.12(0.46)	96.44(0.40)	94.46(0.55)	
**Heart attack**,**%(SE)**						< 0.0001
Yes	5.11(0.00)	3.78(0.37)	4.32(0.45)	5.41(0.56)	7.86(0.70)	
No	94.89(0.02)	96.22(0.37)	95.68(0.45)	94.59(0.56)	92.14(0.70)	
**CHF**,**%(SE)**						< 0.0001
Yes	3.58(0.00)	2.25(0.31)	3.03(0.41)	3.39(0.38)	6.55(0.71)	
No	96.42(0.02)	97.75(0.31)	96.97(0.41)	96.61(0.38)	93.45(0.71)	
**CHD**,**%(SE)**						< 0.001
Yes	5.28(0.00)	4.76(0.49)	4.48(0.51)	4.99(0.60)	7.51(0.69)	
No	94.72(0.02)	95.24(0.49)	95.52(0.51)	95.01(0.60)	92.49(0.69)	
**Angina**,**%(SE)**						0.001
Yes	3.67(0.00)	3.13(0.40)	3.19(0.42)	3.36(0.44)	5.50(0.64)	
No	96.33(0.02)	96.87(0.40)	96.81(0.42)	96.64(0.44)	94.50(0.64)	
**ASCVD**,**%(SE)**						< 0.0001
Yes	11.42(0.01)	9.26(0.62)	10.36(0.74)	11.56(0.88)	15.99(0.95)	
No	88.58(0.02)	90.74(0.62)	89.64(0.74)	88.44(0.88)	84.01(0.92)	

Date are presented as mean (SE) or n (%);PIR: Poverty income ratio, BMI: Body mass index, BUN: Blood urea nitrogen, CVD: Cardiovascular disease, CHF: Congestive heart failure, CHD: Coronary heart disease, ASCVD: Atherosclerotic cardiovascular disease

### Relationship between HGI and cardiovascular disease risk in patients with diabetes or prediabetes

In the fully adjusted Model 3 (Table 2), for each 1-unit increase in HGI, the risk of CVD increases by 1.16 times (OR: 1.16, 95% CI: 1.03–1.30). Meanwhile, the multivariable-adjusted OR and 95% CI for HGI from the lowest to the highest quartiles are 1.00 (reference group), 0.96 (0.77–1.19), 0.90 (0.72–1.12), and 1.32 (1.08–1.63), respectively.

**Table 2:** The relationship between HGI and the risk of cardiovascular diseases in patients with diabetes or prediabetes

Variables	Model 1		Model 2		Model 3	
	OR(95%CI)	*P*	OR(95%CI)	*P*	OR(95%CI)	*P*
**HGI**	1.34(1.20,1.49)	< 0.0001	1.23(1.09,1.40)	< 0.001	1.16(1.03,1.30)	0.02
**HGI Category**						
Q1	Ref	Ref	Ref	Ref	Ref	Ref
Q2	1.14(0.93,1.39)	0.21	0.96(0.77,1.19)	0.69	0.96(0.77,1.19)	0.68
Q3	1.30(1.06,1.60)	0.01	0.95(0.76,1.18)	0.65	0.90(0.72,1.12)	0.34
Q4	1.99(1.65,2.41)	< 0.0001	1.50(1.21,1.85)	< 0.001	1.32(1.08,1.63)	0.01
**P for trend**	< 0.0001		0.008		0.137	

OR: odds ratio, CI: confidence interval, Ref: reference

Model 1: No adjustments made;

Model 2: Adjusted for age, sex, race;

Model 3:Adjusted for age, sex, race, smoking, alcohol, hypertension and BMI;

In the multifactorial analysis of this study, HGI as a continuous variable (increasing by 1 unit) significantly increased the risk of myocardial infarction (OR = 1.23, 95% CI: 1.04–1.44) and the risk of congestive heart failure (OR = 1.31, 95% CI: 1.08–1.59). Further grouping HGI by quartiles revealed that compared to the lowest quartile, the highest quartile HGI group had a 62% increased risk of myocardial infarction (OR = 1.62, 95% CI: 1.18–2.23) and a 97% increased risk of congestive heart failure (OR = 1.97, 95% CI: 1.35–2.88) (Tables 3 and 4).

**Table 3:** The relationship between HGI and the risk of heart attack in patients with diabetes or prediabetes

Variables	Model 1		Model 2		Model 3	
	OR(95%CI)	*P*	OR(95%CI)	*P*	OR(95%CI)	*P*
**HGI**	1.35(1.18,1.54)	< 0.0001	1.33(1.14,1.55)	< 0.001	1.23(1.04,1.44)	0.01
**HGI Category**						
Q1	Ref	Ref	Ref	Ref	Ref	Ref
Q2	1.15(0.85,1.55)	0.37	1.02(0.74,1.40)	0.90	1.00(0.72,1.40)	0.99
Q3	1.45(1.09,1.93)	0.01	1.20(0.90,1.61)	0.21	1.11(0.83,1.48)	0.49
Q4	2.17(1.64,2.86)	< 0.0001	1.90(1.39,2.59)	< 0.0001	1.62(1.18,2.23)	0.003
**P for trend**	< 0.0001		< 0.001		0.006	

OR: odds ratio, CI: confidence interval, Ref: reference

Model 1: No adjustments made;

Model 2: Adjusted for age, sex, race;

Model 3:Adjusted for age, sex, race, smoking, alcohol, hypertension and BMI;

**Table 4:** The relationship between HGI and the risk of congestive heart failure in patients with diabetes or prediabetes

Variables	Model 1		Model 2		Model 3	
	OR(95%CI)	*P*	OR(95%CI)	*P*	OR(95%CI)	*P*
**HGI**	1.50(1.25,1.79)	< 0.0001	1.42(1.15,1.75)	0.001	1.31(1.08,1.59)	0.01
**HGI Category**						
Q1	Ref	Ref	Ref	Ref	Ref	Ref
Q2	1.35(0.93,1.98)	0.12	1.16(0.78,1.72)	0.47	1.18(0.78,1.78)	0.44
Q3	1.52(1.05,2.20)	0.03	1.12(0.76,1.64)	0.56	1.08(0.73,1.58)	0.70
Q4	3.04(2.14,4.32)	< 0.0001	2.24(1.54,3.27)	< 0.0001	1.97(1.35,2.88)	< 0.001
**P for trend**	< 0.0001		< 0.001		0.009	

OR: odds ratio, CI: confidence interval, Ref: reference

Model 1: No adjustments made;

Model 2: Adjusted for age, sex, race;

Model 3:Adjusted for age, sex, race, smoking, alcohol, hypertension and BMI;

In contrast, neither in the analysis as a continuous variable nor in the grouped analysis was there a statistically significant correlation observed between HGI and the risk of other cardiovascular outcomes such as stroke, angina, coronary heart disease (CHD), and atherosclerotic cardiovascular disease (ASCVD) (Tables 5, 6 and 7, and 8).

**Table 5:** The relationship between HGI and the risk of stroke in patients with diabetes or prediabetes

Variables	Model 1		Model 2		Model 3	
	OR(95%CI)	*P*	OR(95%CI)	*P*	OR(95%CI)	*P*
**HGI**	1.29(1.10,1.52)	0.002	1.13(0.92,1.39)	0.24	1.04(0.86,1.26)	0.67
**HGI Category**						
Q1	Ref	Ref	Ref	Ref	Ref	Ref
Q2	1.28(0.88,1.86)	0.19	1.06(0.74,1.52)	0.74	1.05(0.73,1.50)	0.80
Q3	1.17(0.83,1.66)	0.37	0.80(0.57,1.11)	0.18	0.75(0.53,1.05)	0.10
Q4	1.86(1.33,2.60)	< 0.001	1.19(0.83,1.69)	0.34	1.02(0.72,1.44)	0.93
**P for trend**	< 0.001		0.916		0.285	

OR: odds ratio, CI: confidence interval, Ref: reference

Model 1: No adjustments made;

Model 2: Adjusted for age, sex, race;

Model 3:Adjusted for age, sex, race, smoking, alcohol, hypertension and BMI;

**Table 6:** The relationship between HGI and the risk of angina in patients with diabetes or prediabetes

Variables	Model 1		Model 2		Model 3	
	OR(95%CI)	*P*	OR(95%CI)	*P*	OR(95%CI)	*P*
**HGI**	1.26(1.10,1.44)	< 0.001	1.22(1.04,1.42)	0.01	1.12(0.96,1.31)	0.16
**HGI Category**						
Q1	Ref	Ref	Ref	Ref	Ref	Ref
Q2	1.02(0.69,1.52)	0.92	0.90(0.59,1.35)	0.60	0.91(0.60,1.38)	0.64
Q3	1.08(0.76,1.52)	0.67	0.87(0.61,1.23)	0.43	0.82(0.57,1.16)	0.26
Q4	1.80(1.35,2.41)	< 0.0001	1.53(1.13,2.09)	0.01	1.33(0.98,1.82)	0.07
**P for trend**	< 0.001		0.039		0.226	

OR: odds ratio, CI: confidence interval, Ref: reference

Model 1: No adjustments made;

Model 2: Adjusted for age, sex, race;

Model 3:Adjusted for age, sex, race, smoking, alcohol, hypertension and BMI;

**Table 7:** The relationship between HGI and the risk of coronary heart disease in patients with diabetes or prediabetes

Variables	Model 1		Model 2		Model 3	
	OR(95%CI)	*P*	OR(95%CI)	*P*	OR(95%CI)	*P*
**HGI**	1.25(1.08,1.46)	0.003	1.23(1.02,1.49)	0.03	1.18(0.98,1.42)	0.08
**HGI Category**						
Q1	Ref	Ref	Ref	Ref	Ref	Ref
Q2	0.94(0.68,1.30)	0.71	0.80(0.55,1.15)	0.22	0.81(0.56,1.17)	0.26
Q3	1.05(0.78,1.41)	0.73	0.83(0.60,1.13)	0.23	0.79(0.58,1.08)	0.14
Q4	1.62(1.22,2.16)	0.001	1.40(1.01,1.95)	0.04	1.30(0.94,1.79)	0.11
**P for trend**	0.001		0.144		0.32	

OR: odds ratio, CI: confidence interval, Ref: reference

Model 1: No adjustments made;

Model 2: Adjusted for age, sex, race;

Model 3:Adjusted for age, sex, race, smoking, alcohol, hypertension and BMI;

**Table 8:** The relationship between HGI and the risk of atherosclerotic cardiovascular disease in patients with diabetes or prediabetes

Variables	Model 1		Model 2		Model 3	
	OR(95%CI)	*P*	OR(95%CI)	*P*	OR(95%CI)	*P*
**HGI**	1.31(1.17,1.47)	< 0.0001	1.22(1.07,1.39)	0.004	1.14(1.00,1.29)	0.04
**HGI Category**						
Q1	Ref	Ref	Ref	Ref	Ref	Ref
Q2	1.13(0.91,1.41)	0.26	0.96(0.75,1.22)	0.72	0.95(0.74,1.21)	0.68
Q3	1.28(1.02,1.60)	0.03	0.95(0.74,1.21)	0.66	0.89(0.70,1.13)	0.35
Q4	1.87(1.51,2.31)	< 0.0001	1.42(1.11,1.81)	0.01	1.24(0.98,1.57)	0.07
**P for trend**	< 0.0001		0.049		0.363	

OR: odds ratio, CI: confidence interval, Ref: reference

Model 1: No adjustments made;

Model 2: Adjusted for age, sex, race;

Model 3:Adjusted for age, sex, race, smoking, alcohol, hypertension and BMI;

### Association between HGI and risk of cardiovascular disease in patients with diabetes or prediabetes

**Fig. 3:**
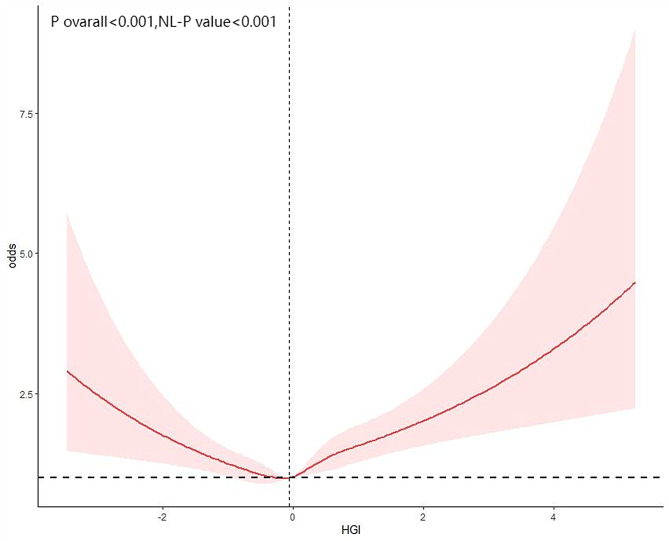
The restricted cubic spline analysis between the HGI and the risk of cardiovascular disease

Restricted cubic spline (RCS) analysis was used to explore the potential nonlinear relationship between HGI and cardiovascular disease (CVD) risk. As shown in Fig. 3, there was a significant nonlinear association between HGI and CVD (P for nonlinearity < 0.0001). Threshold effect analysis further demonstrated a distinct bidirectional (U-shaped) relationship, with the inflection point at HGI = -0.140. Specifically, when baseline HGI was below this threshold, HGI was negatively associated with CVD risk (OR = 0.79, 95% CI: 0.67–0.92); when HGI exceeded the threshold, a positive association with CVD risk was observed (OR = 1.35, 95% CI: 1.23–1.48; Table 9). These results were obtained using segmented multivariable regression, fully adjusted for confounding factors, ensuring the statistical independence and robustness of this nonlinear association.

**Table 9:** Threshold effect analysis of HGI and cardiovascular disease risk in patients with diabetes or Pre-diabetes

	Adjusted OR(95%CI)	*P* value
**Total**	1.15(1.15,1.24)	0.0002
**Segmented cox proportional oddss model**		
**Inflection point**	−0.140	
**HGI**		
HGI<−0.140	0.79(0.67,0.92)	0.003
HGI≥−0.140	1.35(1.23,1.48)	< 0.0001
***P*** **for Log-likehood ratio**		< 0.001

**Fig. 4:**
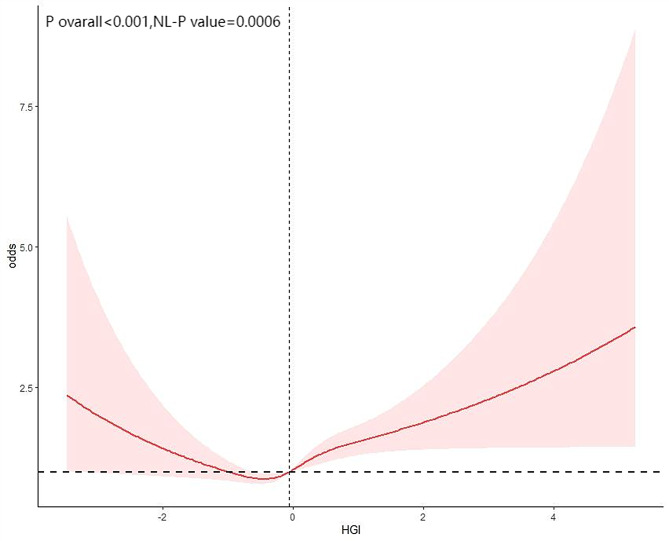
The restricted cubic spline analysis between the HGI and the risk of heart attack

**Fig. 5:**
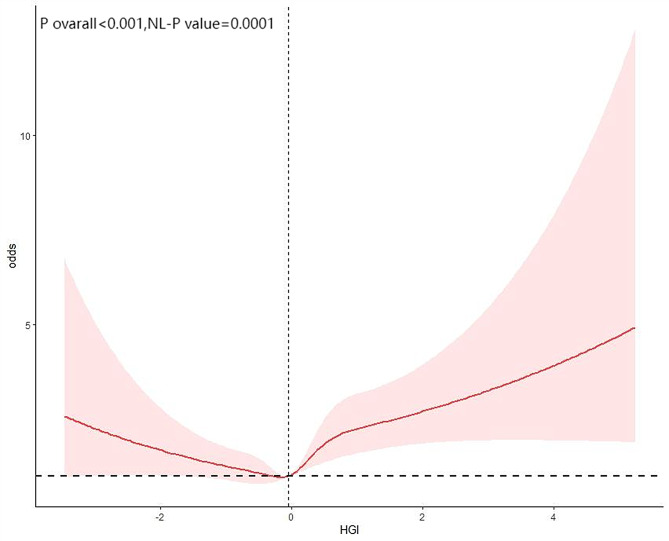
The restricted cubic spline analysis between the HGI and the risk of congestive heart failure

OR: odds ratio, CI: confidence interval;

Adjusted for age, sex, race, smoking, alcohol, hypertension and BMI;

Similarly, Figs. 4 and 5 illustrate the potential nonlinear relationships between HGI and the risks of heart attack and congestive heart failure (CHF). Further analyses revealed significant U-shaped relationships between HGI and heart attack (P for nonlinearity = 0.0006) and CHF (P for nonlinearity = 0.0001). Threshold effect analyses identified inflection points at HGI = -0.447 for heart attack and HGI = -0.140 for CHF. When HGI was below these respective thresholds, negative associations were observed with heart attack (OR = 0.73, 95% CI: 0.57–0.94) and CHF (OR = 0.88, 95% CI: 0.68–1.13); when HGI exceeded these thresholds, positive associations emerged for heart attack (OR = 1.34, 95% CI: 1.20–1.51) and CHF (OR = 1.39, 95% CI: 1.22–1.58; Tables 10 and 11). These findings indicate that the impact of HGI on major cardiovascular outcomes is not unidirectional or simply linear, but rather demonstrates a significant U-shaped risk pattern, which should be interpreted dynamically and stratified in clinical evaluation.

**Table 10:** Threshold effect analysis of HGI and heart attack risk in patients with diabetes or Pre-diabetes

	Adjusted OR(95%CI)	*P* value
**Total**	1.20(1.08,1.34)	0.0008
**Segmented cox proportional oddss model**		
**Inflection point**	−0.447	
**HGI**		
HGI<−0.447	0.73(0.57,0.94)	0.0151
HGI≥−0.447	1.34(1.20,1.51)	< 0.0001
***P*** **for Log-likehood ratio**		< 0.001

OR: odds ratio, CI: confidence interval;

Adjusted for age, sex, race, smoking, alcohol, hypertension and BMI;

**Table 11:** Threshold effect analysis of HGI and congestive heart failure risk in patients with diabetes or Pre-diabetes

	Adjusted OR(95%CI)	*P* value
**Total**	1.25(1.11,1.40)	0.0002
**Segmented cox proportional oddss model**		
**Inflection point**	−0.140	
**HGI**		
HGI<−0.140	0.88(0.68,1.13)	0.307
HGI≥−0.140	1.39(1.22,1.58)	< 0.0001
***P*** **for Log-likehood ratio**		0.007

**Fig. 6:**
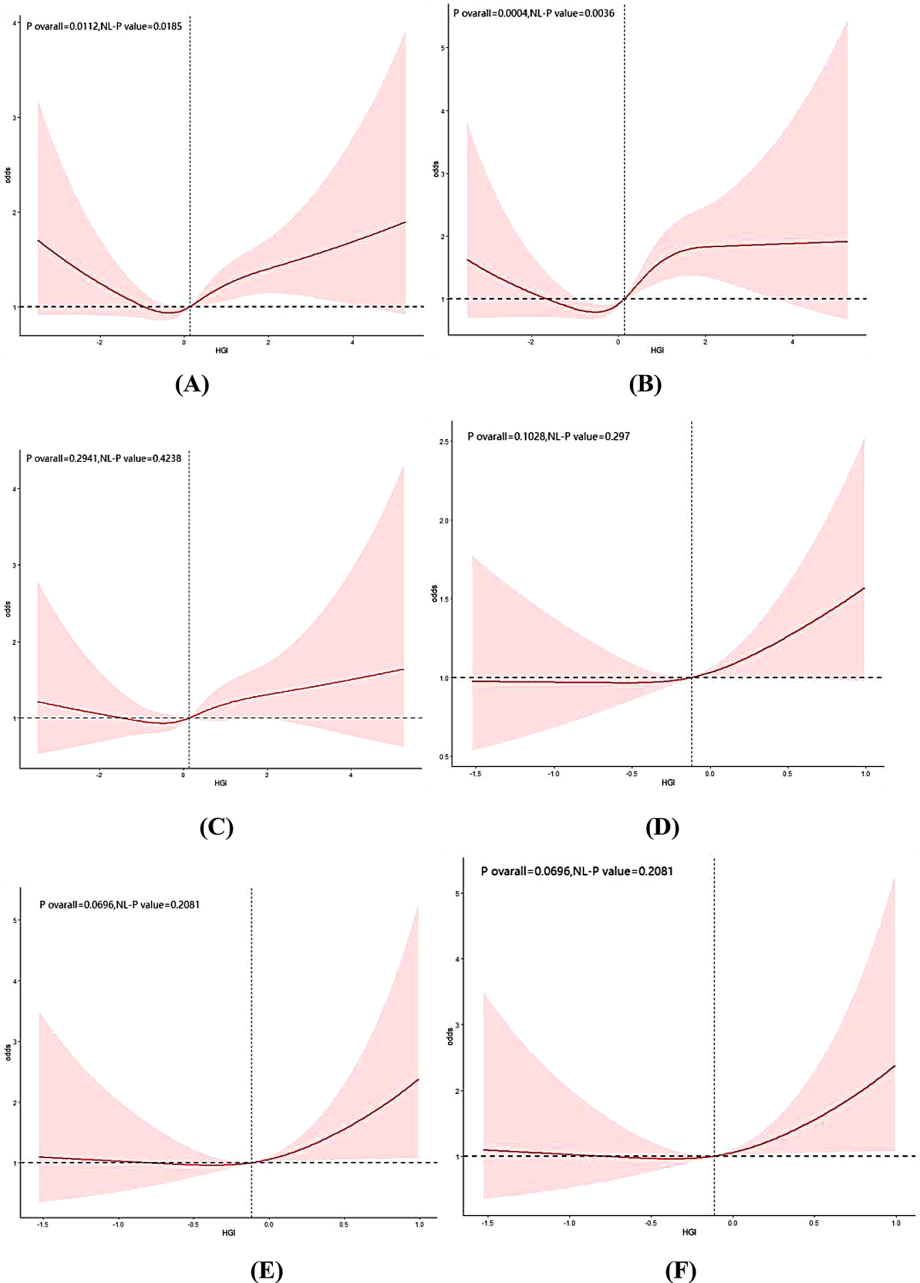
The restricted cubic spline analysis between the HGI and the risk of cardiovascular disease; (**A**) Risk of HGI and CVD in the diabetic population, (**B**) Risk of HGI and heart attack in the diabetic population, (**C**) Risk of HGI and CHF in the diabetic population, (**D**) Risk of HGI and CVD in the prediabetic population, (**E**) Risk of HGI and heart attack in the prediabetic population, (**F**) Risk of HGI and CHF in the prediabetic population

OR: odds ratio, CI: confidence interval;

Adjusted for age, sex, race, smoking, alcohol, hypertension and BMI;

We further investigated the relationship between HGI and vascular disease risk in populations with diabetes and prediabetes (see Fig. 6). The results indicated that in diabetic participants, HGI exhibited a U-shaped relationship with CVD and CHF (nonlinear *P* < 0.001), while it showed a J-shaped relationship with heart attack. Threshold effect analysis revealed that the turning points for HGI with respect to CVD and CHF were − 0.360 and − 0.535, respectively. Specifically, when the baseline HGI is below the turning point, HGI is negatively associated with CVD (OR: 0.81, 95% CI: 0.67–0.99) and CHF (OR: 0.98, 95% CI: 0.70–1.39); when the baseline HGI exceeds the turning point, HGI is positively associated with CVD (OR: 1.19, 95% CI: 1.08–1.31) and CHF (OR: 1.19, 95% CI: 1.05–1.36) (Tables S1 and S2). In individuals with prediabetes, HGI exhibited a J-shaped relationship with CVD, heart attack, and CHF (nonlinear *P* > 0.05) (Fig. 6).

### Subgroup analysis

**Fig. 7:**
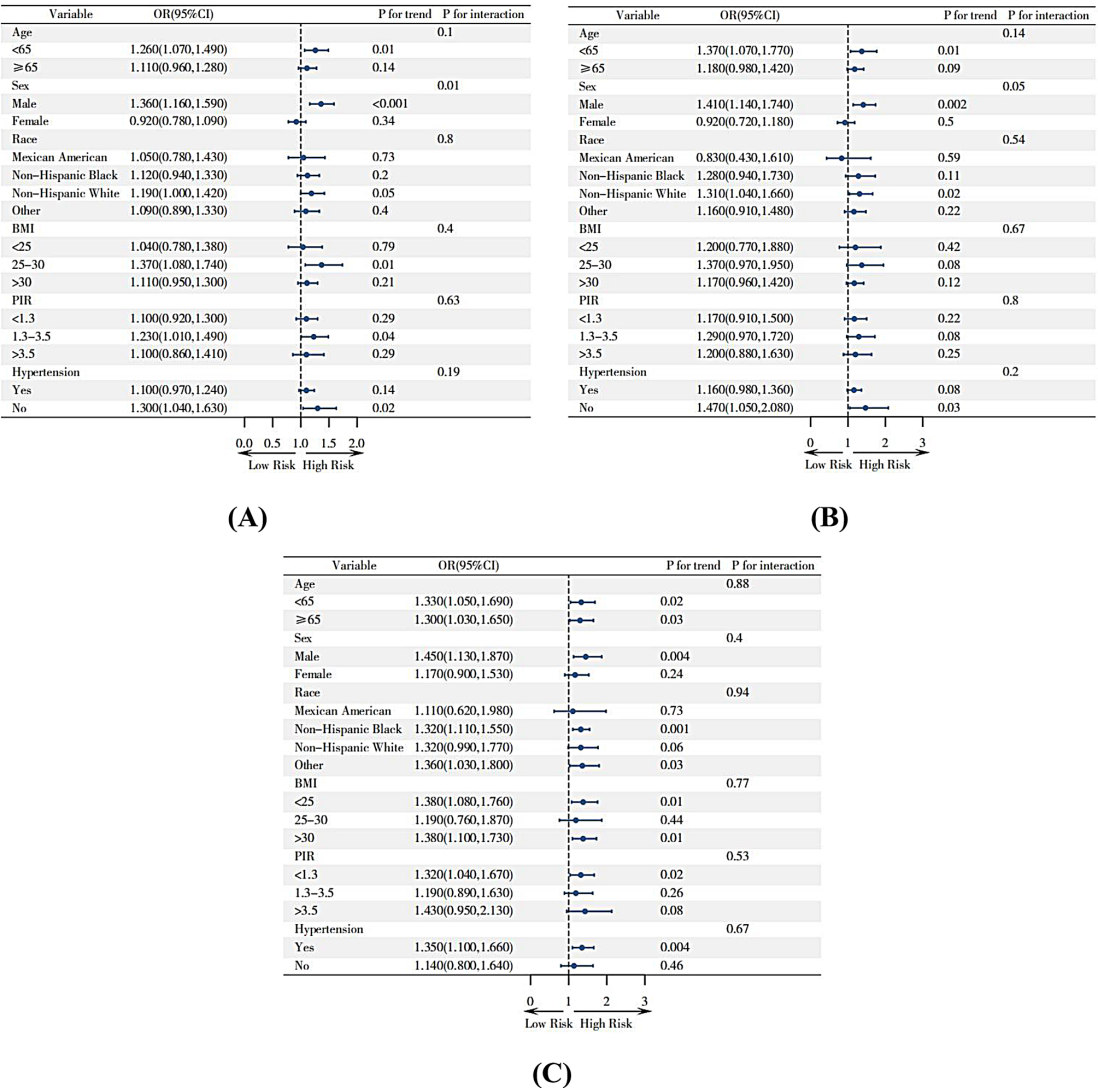
Forest Plot for Subgroup Analyses; (**A**)Subgroup Analyses of HGI and CVD, (**B**)Subgroup Analyses of HGI and heart attack, (**C**)Subgroup Analyses of HGI and CHF

The subgroup analysis in Fig. 7 shows that the relationship between HGI and CVD, as well as heart attack, remains consistent across all subgroups after stratification by age, sex, race, BMI, PIR, and hypertension (interaction *P* > 0.05). However, there is an interaction between baseline HGI and CHF risk in terms of racial stratification (*P* = 0.03), while no significant interactions were found with other stratification variables (*P* > 0.05).

## Discussion

In this study, we explored the relationship between the Hemoglobin Glycation Index (HGI) and the risk of cardiovascular disease (CVD) in individuals with diabetes or prediabetes. We found significant associations between different quartiles of HGI and the prevalence of CVD. Specifically, baseline characteristics of the participants indicated that higher HGI quartiles (Q3 and Q4) were associated with higher CVD prevalence, at 12.60% and 18.08%, respectively, while the lowest quartile (Q1) had a prevalence of only 9.98% [17]. This finding is consistent with previous research, which also indicated that elevated HGI levels are related to an increased risk of cardiovascular disease [36]. For example, some studies suggest that higher HGI may be associated with chronic inflammation and endothelial dysfunction, both of which are significant risk factors for cardiovascular disease [37]. Additionally, consistent with previous research [38], our study also found that high HGI values are more common in certain specific populations, particularly among non-Hispanic Black individuals and women. These associations have been widely reported and reflect the biological differences and potential social environmental influences on glucose metabolism and glycation processes across different populations.

Diabetes and its prediabetic state have become an increasingly severe public health issue globally and are recognized as significant risk factors for cardiovascular diseases (CVD) [39]. The risk of CVD is significantly elevated in diabetic patients due to metabolic disorders, chronic inflammation, and endothelial dysfunction caused by prolonged hyperglycemia [40, 41]. The glycosylated hemoglobin index (HGI) is an important biomarker, and its application in CVD risk assessment has received growing attention [42]. Recent large-scale epidemiological studies, both domestically and internationally, have confirmed a close relationship between elevated HGI and increased CVD risk. For instance, a study by Wang et al., based on NHANES data from the United States, found that an increase in HGI is an independent predictor of elevated CVD risk in the diabetic population, consistent with the findings of this study [42]. Chinese researchers, such as Lin et al., confirmed in diabetic patients with coronary heart disease that HGI elevation can significantly predict the risk of adverse cardiovascular outcomes, indicating that HGI possesses good predictive ability in the Chinese population [19]. Further studies in Korea by Ahn et al. and Lee et al. have shown that high HGI is not only related to adult diabetes but is also closely associated with the increased cardio-metabolic risk factors in non-diabetic adolescents, illustrating the wide applicability of HGI across different populations [43, 44]. A cross-sectional study in Japan also demonstrated that elevated HGI is independently associated with early indicators of CVD, such as arteriosclerosis [45]. On an international scale, a meta-analysis by Wu et al. integrated samples from multiple countries, further proving that high HGI is an important predictor of CVD and all-cause mortality in patients with type 2 diabetes, providing an evidence base for global application [46]. A European study (van Steen et al.) also pointed out that HGI can independently predict the risk of various cardiovascular complications, aside from traditional glycated hemoglobin (HbA1c) levels [47]. These research findings provide substantial validation and supplementation for the conclusions of this study, indicating that HGI is a reliable and applicable tool for predicting cardiovascular disease risk in populations across China, East Asia, and Europe and North America. This study further confirms the importance of HGI in CVD risk prediction, finding that an increase of 1 unit in HGI corresponds to a 1.16-fold increase in CVD risk, which aligns with the aforementioned research results. Furthermore, elevated HGI is closely associated with other cardiovascular risk factors such as blood lipids, blood pressure, and body mass index [48, 49], which further supports its potential as a comprehensive risk assessment tool. Additionally, different quartiles of HGI are significantly related to the risk of heart attack and congestive heart failure (CHF) [50, 51]. This finding emphasizes the importance of monitoring HGI in diabetes management. By timely identifying high-risk patients, clinicians can implement appropriate interventions, such as lifestyle modifications and adjustments to medical treatment, thereby reducing the incidence of cardiovascular events.

It is noteworthy that this study utilized Restricted Cubic Spline (RCS) curve analysis and found a nonlinear relationship between HGI and CVD risk. Similar phenomena have been reported in some cohort studies in China and Europe, suggesting that the clinical significance of HGI in risk stratification and early intervention needs to be interpreted flexibly based on the characteristics of different populations [52]. Subgroup analyses also revealed that the associations between HGI and CVD as well as the risk of heart attack were consistent across various age groups, genders, weights, income levels, and hypertension statuses, indicating that HGI has good generalizability across diverse populations. However, in terms of CHF risk, a significant interaction was found between HGI and race (*P* = 0.03), which is in line with findings from some international studies regarding the influence of genetic and population differences [53]. These findings not only provide new insights for the early prevention and individualized management of CVD, but also indicate that when promoting HGI-related screening and interventions in different countries and regions, it is essential to consider the specific genetic and environmental backgrounds of populations to develop more reasonable health management strategies. Future research should further explore the value of combining HGI with other biomarkers to provide stronger scientific evidence for precise prevention and treatment.

In addition to the positive relationship between HGI and cardiovascular disease risk observed in this study and most epidemiological literature, it is noteworthy that recent large international randomized controlled trials have reported uncertainties and controversies regarding the predictive ability of HGI. For example, in the ADVANCE study, patients with high HGI in the intensified glycemic therapy group exhibited a lower all-cause mortality risk than expected [54]. The authors suggest that intensive diabetes management may offset some of the adverse metabolic impacts experienced by patients with high HGI. In contrast, the ACCORD study found that high HGI patients in the intensive treatment group had a significantly increased risk of mortality [11]. These inconsistent conclusions indicate that the clinical significance of HGI is quite complex, influenced by multiple factors such as treatment regimens, ethnicity, and baseline cardiovascular risk, and may also reflect the heterogeneity of metabolic pathways associated with HGI.

The clinical predictive value of HGI may vary significantly among different populations and disease states. Studies have shown that HGI is not only associated with diabetes and its complications but may also be influenced by individual patient characteristics, underlying conditions (such as renal function, anemia status, etc.), and treatment regimens. Research focused on patients with acute decompensated heart failure (ADHF) indicates that the prognostic role of HGI in this specific population is significantly different from findings in diabetic or general populations. In ADHF patients, HGI does not consistently demonstrate predictive ability for cardiovascular outcomes, suggesting that its clinical applicability in specific disease states is limited [55]. This reflects a certain degree of heterogeneity in populations when considering HGI as a biomarker, indicating that its predictive significance should not be simply extrapolated to all patients or clinical contexts.

As a biomarker, HGI has the following limitations:①it is fundamentally a mathematical residual, closely related to the measurement methods of HbA1c and blood glucose, as well as individual metabolic differences, making it susceptible to confounding factors such as red blood cell lifespan and liver and kidney function;②there is currently no recognized standard for the optimal risk threshold for HGI or its predictive value in dynamic changes;③the causal relationship between HGI and endpoint events in different populations lacks direct verification through interventional trials; ④some studies have shown that the clinical significance of HGI varies significantly under intensive glycemic control and conventional glycemic control, suggesting that clinical interpretations should be made in conjunction with the specific treatment context.

It is important to note that HGI is a parameter derived from the regression calculation based on HbA1c and fasting blood glucose levels; therefore, it has an inherent correlation with these two fundamental blood glucose indicators. The current study observed a certain association between HGI and the risk of some macrovascular complications, but this does not imply that its clinical value as an independent predictor is definitively established. Given that HGI reflects the differences between individuals’ blood glucose levels and glycemic outcomes, its potential value lies in revealing the risks of metabolic heterogeneity in patients that conventional glucose indicators cannot fully capture. However, whether HGI can provide additional predictive capability over individual indicators such as HbA1c still requires further direct comparative analysis for validation.

Therefore, HGI is currently more suited to serve as an auxiliary risk assessment tool to help identify high-risk subgroups that are difficult to delineate with conventional indicators, but it cannot simply replace the clinical significance of HbA1c. Future studies should involve larger samples, multicenter designs, and follow-up research to systematically compare the independence and complementarity of HGI and HbA1c in predicting macrovascular outcomes, thus scientifically evaluating its clinical application value.

The main strength of this study lies in its analysis of data from 10,889 patients with diabetes or pre-diabetes based on the NHANES database, which provides a large and nationally representative sample. This large-scale sample analysis enhances the statistical power and external validity of the study’s results, making the conclusions more broadly applicable. However, there are also some limitations in this study. Firstly, as a cross-sectional study, it cannot determine the causal relationship between HGI and cardiovascular disease. Although significant associations were found, it is not possible to exclude reverse causality or the influence of other unmeasured factors. Secondly, despite using NHANES data, there may be biases in sample selection, particularly regarding the representativeness of patients with diabetes or pre-diabetes, which could affect the generalizability of the results.Thirdly, although the study conducted multivariable adjustments, there may still be unmeasured confounding factors (such as dietary habits, medication use, stress, and family history) that could influence the relationship between HGI and cardiovascular disease risk. It is recommended that future multicenter prospective studies supplement the pertinent information to enhance the comprehensive assessment of disease risk.Fourthly, the study primarily focused on HGI and did not consider other potential biomarkers that may affect cardiovascular disease risk, such as inflammatory markers or lipid levels, which could lead to an underestimation or overestimation of the role of HGI. Fifthly, subgroup analyses indicated significant differences in the risk of CHF associated with HGI across different racial groups, but the study did not delve deeper into the potential mechanisms behind these racial differences. This limitation restricts the applicability of the results to different racial populations. Sixthly, this study relies partially on random blood glucose results for the classification of diabetes and prediabetes due to the absence of acute hyperglycemic symptoms related to diabetes in the NHANES database. This limitation may lead to a certain risk of misclassification, and the study conclusions should be interpreted cautiously in light of the characteristics of the database.Finally, HGI is based on a single measurement, which does not reflect the impact of its dynamic changes on cardiovascular disease risk. Future research should consider longitudinal designs to assess the relationship between changes in HGI over time and cardiovascular disease risk.

Our findings indicate a potential association between HGI and cardiovascular risk, suggesting that HGI may serve as a supplementary marker in diabetes and prediabetes populations. However, given the exploratory nature of these results and the lack of consistent evidence from large-scale studies, further research is needed to clarify the clinical utility of HGI and to determine its role.

## Electronic supplementary material

Below is the link to the electronic supplementary material.

Supplementary Material 1

Supplementary Material 2

## Data Availability

Data are accessible via a public repository with open access. On the NHANES website, data are provided with open access. https://www.cdc.gov/nchs/nhanes/index.htm.
