# Supplementary material for: The relationship between hemoglobin glycation index and the risk of cardiovascular disease in populations with diabetes or prediabetes: a population-based cohort study
Source: Diabetol Metab Syndr. 2025 May 26;17:171. doi: 10.1186/s13098-025-01754-0 (PMC12105251; doi:10.1186/s13098-025-01754-0)
Supplement: Supplementary file 1 — Supplementary Material 1 [file 13098_2025_1754_MOESM1_ESM.doc]

**The relationship between hemoglobin glycation index and the risk of cardiovascular disease in populations with diabetes or prediabetes: A population-based cohort study**

Zheng Wang^1,*,+^,Fachao Shi^2,+^,Long Wang,Caoyang Fang^3,*^

^1^Department of Cardiology, The Second People's Hospital of Hefei, Hefei Hospital Affiliated to Anhui Medical University, Hefei, Anhui 230000, China;

^2^Department of Cardiology,Maanshan People's Hospital, Maanshan, Anhui 243000, China;

^3^Department of Emergency,First Affiliated Hospital of University of Science and Technology of China,Anhui Provincial Hospital, Hefei, Anhui,230000, China;

***Corresponding Author:**

Zheng Wang,Department of Cardiology, The Second People's Hospital of Hefei, Hefei Hospital Affiliated to Anhui Medical University, Hefei, Anhui 230000, China;

Caoyang Fang,Department of Emergency,First Affiliated Hospital of University of Science and Technology of China,Anhui Provincial Hospital, Hefei, Anhui,230000, China;

^+^:They contributed equally to the article.

**Supplementary Table 1.Threshold Effect Analysis of HGI and cardiovascular diseases Risk in Patients with Pre-diabetes**

|  | **Adjusted OR(95%CI)** | ***P* value** |
| --- | --- | --- |
| **Total** | 1.08(1.00,1.17) | 0.0456 |
| **Segmented cox proportional oddss model** |  |  |
| **Inflection point** | -0.360 |  |
| **HGI** |  |  |
| HGI<-0.360 | 0.81(0.67,0.99) | 0.0381 |
| HGI≥-0.360 | 1.19(1.08,1.31) | 0.0005 |
| ***P* for Log-likehood ratio** |  | 0.002 |

**OR: odds ratio, CI: confidence interval;**

**Adjusted for age, sex, race,smoking, alcohol, hypertension and BMI;**

**Supplementary Table 2.Threshold Effect Analysis of HGI and congestive heart failure Risk in Patients with Pre-diabetes**

|  | **Adjusted OR(95%CI)** | ***P* value** |
| --- | --- | --- |
| **Total** | 1.16(1.03,1.30) | 0.0149 |
| **Segmented cox proportional oddss model** |  |  |
| **Inflection point** | -0.535 |  |
| HGI |  |  |
| HGI<-0.535 | 0.98(0.70,1.39) | 0.9281 |
| HGI≥-0.535 | 1.19(1.05,1.36) | 0.009 |
| ***P* for Log-likehood ratio** |  | 0.365 |

**OR: odds ratio, CI: confidence interval;**

**Adjusted for age, sex, race,smoking, alcohol, hypertension and BMI;**
